# Prevalencia de anomalías dentarias de tamaño y forma, en pacientes pediátricos de 6 a 17 años de la ciudad de huánuco, 2019-2022

**DOI:** 10.21142/2523-2754-1104-2023-171

**Published:** 2023-12-28

**Authors:** Claudia Palacios León, Carol Cárdenas Flores

**Affiliations:** 1 Posgrado de Odontopediatría, Universidad Científica del Sur. Lima, Perú. 100026364@cientifica.edu.pe Universidad Científica del Sur Posgrado de Odontopediatría Universidad Científica del Sur Lima Peru 100026364@cientifica.edu.pe; 2 Carrera de Estomatología, Universidad Científica del Sur. Lima, Perú., ccardenasf@cientifica.edu.pe Universidad Científica del Sur Carrera de Estomatología Universidad Científica del Sur Lima Peru ccardenasf@cientifica.edu.pe

**Keywords:** anomalías dentarias, anomalía de número, anomalía de tamaño, radiografía panorámica, prevalencia, dental anomalies, number anomaly, size anomaly, panoramic radiography, prevalence

## Abstract

**Objetivo:**

Determinar la prevalencia de anomalías dentales de tamaño y forma en pacientes de 6 a 17 años, atendidos en Huánuco entre 2019 y 2022.

**Metodología:**

El estudio fue de tipo descriptivo, de corte transversal retrospectivo, con una muestra de 362 radiografías panorámicas de pacientes de 6 a 17 años. Se realizó observaciones detalladas para cada ficha de observación: la edad del paciente, el sexo, las alteraciones dentarias de forma y tamaño, el tipo de dentición que presentaba, el cuadrante afectado y el tipo de pieza dentaria.

**Resultados:**

Se encontró una mayor prevalencia de anomalías dentarias de forma, con el 85,10% (308), y de tamaño, con un 14,9% (54). En relación con el cuadrante, el que presentó mayor prevalencia fue el diente con rizomicri, con el 18,5% (10) en el cuadrante II, y la macrodoncia, con el 13,0% (7) en los cuadrantes I y II; mientras que la alteración de forma diente de pala presentó una mayor prevalencia en el cuadrante II, con el 24,5% (76).

**Conclusión:**

Existe mayor prevalencia de anomalías de forma, a diferencia de las anomalías de tamaño, que fueron menos prevalentes en el trabajo de investigación, y fueron el sexo femenino y el grupo etario de 11 a 17 años los más prevalentes.

## Abstract (en)

**Objective:**

To determine the prevalence of dental anomalies of size and shape in patients aged 6 to 17 years, treated in Huánuco from 2019 to 2022.

**Methodology:**

The study was descriptive, retrospective cross-sectional with a sample of 362 Panoramic radiographs of patients 6 to 17 years of age. I make detailed observations for each observation sheet, the patient's age, sex, dental shape and size alterations, the type of dentition presented, the affected quadrant and the type of dental piece.

**Results:**

A higher prevalence of dental anomaly of shape was found with 85.10% (308) and of size with 14.9% (54). In relation to the quadrant, the tooth with rhizomycorrhexis presented the highest prevalence with 18.5% (10) in quadrant II while macrodontia presented 13.0% (7) in quadrant I and II, while the shape alteration of the shovel tooth presented the highest prevalence in quadrant II with 24.5% (76).

**Conclusion:**

There is a higher prevalence of shape anomalies as opposed to size anomalies that were less prevalent in the research work, being the female sex and the age group 11 to 17 years the most prevalent.

## INTRODUCCIÓN

Las anomalías dentales son alteraciones que pueden afectar el crecimiento de los dientes en términos de cantidad, dimensión y morfología. Estas surgen debido a varios factores que incluyen condiciones sistémicas, influencias ambientales, factores locales y rasgos hereditarios ^2^^,^^3^. Las anomalías en la morfología dañan tanto el esmalte tanto como la dentina ^4^, lo que provoca alteraciones estéticas y funcionales ^5^.

El enfoque principal del estudio se centró en las patologías dentales de forma y tamaño. La anomalía de forma se definirá como resultado de factores complejos, derivados de cambios patológicos que ocurren durante el crecimiento embrionario del diente ^6^^,^^7^. El crecimiento insuficiente durante la etapa morfológica de la geminación del diente es responsable de la anomalía en cuestión. Cabe señalar que, en esta anomalía en particular, la morfología del diente se mantiene normal y sin cambios; la única diferencia perceptible es el tamaño ^8^^-^^11^. 

La importancia del análisis radiográfico radicó en su capacidad para evaluar las diversas patologías descubiertas dentro de la cavidad oral, incluida la alteración de forma, tamaño, entre otras patologías ^12^^-^^15^. Al ser la radiografía panorámica un examen complementario, también ayuda al odontólogo a realizar un diagnóstico final para una correcta planificación del tratamiento, de acuerdo con los diferentes cambios patológicos del paciente ^16^^-^^18^.

En una investigación realizada por Cordero ^19^ se encontró que la anomalía de tamaño más observada era la macrodoncia, y que los dientes más afectados fueron los caninos y los incisivos laterales inferiores. En el estudio realizado por Alfaro ^20^, se descubrió que, cuando se trata de anomalías de forma, la mayor prevalencia se da en el sexo masculino y predomina el cuadrante IV. Por el contrario, en cuanto a las desviaciones de tamaño, el género femenino muestra una mayor ocurrencia, con predominio de los cuadrantes I y II.

Como especialistas en odontopediatría, nos hemos encontrado con un número creciente de anomalías dentales a lo largo del tiempo. Es nuestra responsabilidad investigar la prevalencia de estos trastornos del desarrollo, ya que tienen un impacto significativo en la estética y funcionalidad de los dientes de nuestros pacientes. Por lo tanto, enfatizamos la importancia de priorizar las radiografías de diagnóstico para ayudar en la planificación del tratamiento y minimizar los desafíos inesperados durante el curso del tratamiento.

El objetivo de la investigación fue determinar la frecuencia de anomalías en el tamaño y la forma de dientes primarios y permanentes en radiografía panorámica, en niños con edades entre 6 y 17 años, que visitaron dos centros de diagnóstico por imágenes en la ciudad de Huánuco, entre los años 2019 y 2022.

## MATERIALES Y MÉTODOS

La investigación es de tipo descriptivo, transversal y retrospectiva, y fue aprobada por el Comité de Investigación y Ética de la Universidad Científica del Sur con el numero POS-94-2022-00152. Se utilizó como método la observación para recopilar los datos de las radiografías. La población la conformaron 1080 radiografías panorámicas de pacientes con edades entre 6 y 17 años, que acudieron a los centros radiológicos Cero y Cedident Digital, de la ciudad de Huánuco, de enero de 2019 a setiembre de 2022. La muestra se obtuvo mediante un muestreo probabilístico aleatorio simple, en el cual se aplicó la fórmula finita y se obtuvo 362 radiografías panorámicas. 

Se consideraron como criterios de inclusión las radiografías panorámicas de pacientes de 6 a 17 años de ambos géneros, así como las radiografías panorámicas con buen contraste. Se excluyeron las radiografías panorámicas con un contraste distorsionado, radiografías de pacientes con aparatología fija o removible de ortodoncia, radiografías panorámicas de pacientes con antecedentes de síndromes, radiografías panorámicas de pacientes con paladar fisurado, y radiografías del mismo paciente.

Se dispuso una herramienta modificada y validada por la especialista en radiología. Esta hoja describe el tipo, la posición, el sexo y la edad de las anomalías dentales. Después, se eligieron las imágenes radiográficas digitales. Este proceso de selección se realizó en una PC con procesador Core i7. Tras la selección de la muestra de estudio, las radiografías fueron evaluadas con el instrumento de recolección de datos. 

Se evaluó, en un ambiente oscuro, un promedio de 12 radiografías por día, divididas en 6 radiografías por la mañana y 6 radiografías por la tarde. Para el análisis de cada radiografía se tomó un tiempo de 20 minutos, iniciando del cuadrante I al IV en orden, y tomándose 10 minutos de descanso por radiografía. 

A fin de examinar y evaluar las anomalías en forma y tamaño dental, se utilizó el *software* radiográfico EasyDent, el cual proporcionó los medios para identificar los tipos específicos de patologías, incluida la macrodoncia, que se observó radiográficamente como una pieza dental más grande de lo normal; la microdoncia, con una pieza dental más pequeña de lo normal; la rizomegalia, observada radiológicamente como un alargamiento de las piezas dentarias; y la rizomicri, caracterizada por presentar las raíces de la pieza dental más pequeñas de lo normal. La observación de la arcada más prevalente se realizó utilizando la herramienta del programa EasyDent, que facilitó la división en cuatro cuadrantes. Además, se realizó una evaluación para determinar la pieza dentaria más impactada por esta condición en particular. 

Para las variables características sexo y edad, los datos del estudio se obtuvieron mediante el programa EasyDent. Una vez recopilada toda la información requerida, se trasladó al *software* Excel. Posteriormente, los datos se transfirieron al programa estadístico SPSS V26, con el fin de tabularlos y analizarlos. Después de realizar el análisis, los resultados recogidos fueron sometidos a examen mediante el uso de estadísticas descriptivas. 

## RESULTADOS

De las 362 radiografías que conformaron la muestra, el 28,7% (104) pertenecieron a niños de 6 a 10 años, y el 71,3% (258), a niños de 11 a 17 años. Se encontró una mayor prevalencia de anomalía dentaria de forma, con el 85,10% (308), y de tamaño, con un 14,9% (54). Al observar la prevalencia de las anomalías dentarias de tamaño según el cuadrante, se halló que existe una prevalencia de macrodoncia, con el 13,0% ^7^ en el cuadrante I y II, mientras que en el cuadrante III y IV no se reportaron casos. La rizomegalia se encontró en los cuadrantes I y II un 9,3% ^5^, y en los cuadrantes III y IV, el 5,6% ^3^; mientras que el tamaño de tipo rizomicri, se encontró en el cuadrante I un 14,8% ^8^; en el cuadrante II, un 18,5% ^10^; en el cuadrante III, un 7,4% ^4^, y en el cuadrante IV, un 3,7% ^2^ (Tabla 1).

**Tabla 1:** Cuadrante de los maxilares con mayor prevalencia de las anomalías dentarias de tamaño

			Cuadrante				Total
			Cuadrante I	Cuadrante II	Cuadrante III	Cuadrante IV	
Tamaño	Macrodoncia	n	7	7	0	0	14
		%	13,0%	13,0%	0,0%	0,0%	25,9%
	Rizomegalia	n	5	5	3	3	16
		%	9,3%	9,3%	5,6%	5,6%	29,6%
	Rizomicri	n	8	10	4	2	24
		%	14,8%	18,5%	7,4%	3,7%	44,4%
Total		n	20	22	7	5	54
		%	37,0%	40,7%	13,0%	9,3%	100,0%

Se observó la frecuencia de la anomalía dentaria de forma según el cuadrante vemos que existe una prevalencia mayor de la fusión de 1,9% ^6^, en el cuadrante IV. La geminación presentó una prevalencia del 1,3% ^4^ en el cuadrante IV. El diente clavija presentó una prevalencia del 1,0% ^3^ en el cuadrante I y II, y el taurodontismo presentó una prevalencia del 5,5% ^17^ en el cuadrante II. El diente invaginado presentó una frecuencia del 1,3% ^4^ en el cuadrante II. El diente evaginado presentó una prevalencia del 7,1% ^(22)^ en el cuadrante II. El diente en pala presentó una prevalencia del 24,5% (76) en el cuadrante II. El diente dilacerado presentó una prevalencia del 2,9% ^9^ para el cuadrante I. Los dientes con raíces supernumerarios presentaron una prevalencia del 3,5% ^11^ en el cuadrante II. Los dientes con reducción del número de raíces presentaron una prevalencia del 1,3% ^4^ en los cuadrantes I, II y IV (Tabla 2).

**Tabla 2:** Cuadrante de los maxilares con mayor prevalencia de las anomalías dentarias de forma

			Cuadrante				Total
			Cuadrante I	Cuadrante II	Cuadrante III	Cuadrante IV	
Forma	Fusión	n	4	4	0	6	14
		%	1,3%	1,3%	0,0%	1,9%	4,5%
	Geminación	n	0	0	0	4	4
		%	0,0%	0,0%	0,0%	1,3%	1,3%
	Diente clavija	n	3	3	0	0	6
		%	1,0%	1,0%	0,0%	0,0%	1,9%
	Taurodontismo	n	14	17	2	2	35
		%	4,5%	5,5%	0,6%	0,6%	11,3%
	Diente invaginado	n	3	4	0	0	7
		%	1,0%	1,3%	0,0%	0,0%	2,3%
	Diente evaginado	n	19	22	0	0	41
		%	6,1%	7,1%	0,0%	0,0%	13,2%
	Diente en pala	n	74	76	4	0	154
		%	23,9%	24,5%	1,3%	0,0%	49,7%
	Dilaceración	n	9	5	6	0	20
		%	2,9%	1,6%	1,9%	0,0%	6,5%
	Raíces supernumerarias	n	6	11	0	0	17
		%	1,9%	3,5%	0,0%	0,0%	5,5%
	Reducción del número de raíces	n	4	4	0	4	12
		%	1,3%	1,3%	0,0%	1,3%	3,9%
Total		n	136	146	12	16	310
		%	43.9%	47,1%	3,9%	5,2%	100,0%

Se observó la prevalencia de las anomalías dentarias de tamaño según el tipo de dentición permanente. Existe una prevalencia de macrodoncia, con el 25,9% ^14^; la rizomegalia presentó una prevalencia del 29,6% ^16^ y el tamaño de tipo rizomicri mostró una prevalencia del 44,4% ^24^. En la dentición decidua no se reportaron casos de anomalías de tamaño (Tabla 3). En el caso de las anomalías dentarias de forma según el tipo de dentición vemos que existe una prevalencia en la dentición permanente, y la más frecuente es el diente en pala, con el 49,7% (Tabla 3).

**Tabla 3:** Tipo de dentición con mayor prevalencia de anomalías dentarias de tamaño y forma

			Tipo de dentición		Total
			Decidua	Permanente	
Tamaño	Macrodoncia	n		14	14
		%		25,9%	25,9%
	Rizomegalia	n		16	16
		%		29,6%	29,6%
	Rizomicri	n		24	24
		%		44,4%	44,4%
	Total	n		54	54
		%		100,0%	100,0%
Forma	Fusión	n	2	12	14
		%	0,6%	3,9%	4,5%
	Geminación	n	2	2	4
		%	0,6%	0,6%	1,3%
	Diente clavija	n	0	6	6
		%	0,0%	1,9%	1,9%
	Taurodontismo	n	0	35	35
		%	0,0%	11,3%	11,3%
	Diente invaginado	n	0	7	7
		%	0,0%	2,3%	2,3%
	Diente evaginado	n	0	41	41
		%	0,0%	13,2%	13,2%
	Diente en pala	n	0	154	154
		%	0,0%	49,7%	49,7%
	Dilaceración	n	0	20	20
		%	0,0%	6,5%	6,5%
	Raíces supernumerarias	n	0	17	17
		%	0,0%	5,5%	5,5%
	Reducción del número de raíces	n	0	12	12
		%	0,0%	3,9%	3,9%
Total		n	4	306	310
		%	1.3%	98,7%	100,0%

Se observó la prevalencia de la anomalía dentaria de tamaño según el tipo de pieza dentaria. Existe una prevalencia de la macrodoncia, con el 3,7% ^2^ en el incisivo central, 13,0% ^7^ en el incisivo lateral y 9,3% ^5^ en el segundo molar. La rizomegalia presentó una prevalencia del 29,6% ^16^ en el tipo canino, y el tamaño de tipo rizomicri mostró una prevalencia del 7,4% ^4^ en el incisivo central, el 3,7% ^2^ en el incisivo lateral, el 11,1% ^6^ en el primer premolar, y el 22,2% ^12^ en el segundo premolar. En el caso de las anomalías dentarias de forma según la pieza dentaria se puede observar que la mayoría afectan principalmente el incisivo lateral (Tabla 4).

**Tabla 4:** Tipo de pieza dental con mayor prevalencia de anomalías dentarias de forma

			Tipo de pieza dental						Total
			Incisivo central	Incisivo lateral	Canino	Primer premolar	Primer molar	Segundo molar	
Tamaño	Macrodoncia	n	2	7	0	0	0	5	14
		%	3,7%	13,0%	0,0%	0,0%	0,0%	9,3%	25,9%
	Rizomegalia	n	0	0	16	0	0	0	16
		%	0,0%	0,0%	29,6%	0,0%	0,0%	0,0%	29,6%
	Rizomicri	n	4	2	0	6	12	0	24
		%	7,4%	3,7%	0,0%	11,1%	22,2%	0,0%	44,4%
	Total	n	6	9	16	6	12	5	54
		%	11,1%	16,7%	29,6%	11,1%	22,2%	9,3%	100,0%
Forma	Fusión	n	6	6	2	0	0	0	14
		%	1,9%	1,9%	0,6%	0,0%	0,0%	0,0%	4,5%
	Geminación	n	2	2	0	0	0	0	4
		%	0,6%	0,6%	0,0%	0,0%	0,0%	0,0%	1,3%
	Diente clavija	n	0	6	0	0	0	0	6
		%	0,0%	1,9%	0,0%	0,0%	0,0%	0,0%	1,9%
	Taurodontismo	n	0	0	0	0	24	11	35
		%	0,0%	0,0%	0,0%	0,0%	7,7%	3,5%	11,3%
	Diente invaginado	n	0	5	2	0	0	0	7
		%	0,0%	1,6%	0,6%	0,0%	0,0%	0,0%	2,3%
	Diente evaginado	n	10	25	6	0	0	0	41
		%	3,2%	8,1%	1,9%	0,0%	0,0%	0,0%	13,2%
	Diente en pala	n	74	80	0	0	0	0	154
		%	23,9%	25,8%	0,0%	0,0%	0,0%	0,0%	49,7%
	Dilaceración	n	3	2	4	7	4	0	20
		%	1,0%	0,6%	1,3%	2,3%	1,3%	0,0%	6,5%
	Raíces supernumerarias	n	0	8	0	3	0	6	17
		%	0,0%	2,6%	0,0%	1,0%	0,0%	1,9%	5,5%
	Reducción del número de raíces	n	0	0	0	0	2	10	12
		%	0,0%	0,0%	0,0%	0,0%	0,6%	3,2%	3,9%
Total		n	95	134	14	12	30	27	310
		%	30,6%	42,8%	4,5%	3,9%	9,7%	8,7%	100,0%

## DISCUSIÓN

Zapata ^21^ encontró una baja prevalencia de alteraciones, con una incidencia inferior al 1%. Estas anomalías incluyen invaginados, dilacerados, traslaciones, *dens in dent*, macrodoncia y diente conoide. De estos, la macrodoncia es la más prevalente, particularmente en las piezas 4.1 y 3.1. Además, existen anomalías con una incidencia inferior al 1%, como invaginaciones y dilaceraciones. Estos resultados no guardan relación con el presente estudio, ya que en la investigación demostramos la alta incidencia de anomalías dentarias de forma, con un 85,10% (308), y con menor frecuencia las anomalías de tamaño, con un 14,9% (54). Asismimo, la pieza con la anomalía más frecuente fue la del diente en pala, que se presentó en los incisivos en un 49,7%.

Bernardo *et al*. ^22^ evaluaron 300 radiografías de pacientes de 12 a 18 años de edad. Se descubrió que las irregularidades dentales aparecen con mayor frecuencia en el sexo masculino, con una prevalencia del 58,1%. Las mujeres, por su parte, exhibieron una prevalencia del 41,9%. La anomalía dentaria de tamaño más frecuente fue la microdoncia, con una frecuencia del 1,3%. Las dos anomalías de forma más frecuentes fueron la dilaceración y la invaginación, con tasas del 4,3% y el 4%, respectivamente. La anomalía dentaria que se presentó con mayor frecuencia en la erupción dentaria fueron los dientes impactados, que representaron el 22% de los casos. Estos resultados difieren con los de este estudio respecto del sexo, pues se presentó una mayor prevalencia de anomalías de tamaño y forma en el sexo femenino, con un 53,0% (192), mientras que el sexo masculino presentó una prevalencia del 47,0% (170).

Palacios ^23^ encontró que el 61,5% de los individuos presentaban anomalías dentales. En particular, el maxilar inferior tuvo la mayor incidencia de anomalías, con un 37,9%, seguido de cerca por el maxilar superior, que exhibió una prevalencia del 31,3%. Cuando se hizo el análisis por grupo de edad, se reveló que la prevalencia de AD fue del 6,05% entre niños, 15,24% entre adolescentes, 25,13% entre adultos jóvenes y 14,97% entre adultos. La dilaceración es la anomalía más frecuente, con una prevalencia del 25,95%, seguida por la hipodoncia, con el 7,22%, y la hiperdoncia, con el 6,88%. Otras anomalías incluyeron microdoncia (4,01%), macrodoncia (0,80%), concrescencia (0,27%), taurodontismo (10,70%), *dens in dente* (1,87%), conoide (2,41%) y evaginado (0,27%). Estos resultados muestran diferencias con el presente estudio, debido a que el diente en pala presentó mayor prevalencia, con el 49,7%. El autor señala una discrepancia en sus hallazgos, ya que encontraron que la alteración más frecuente de la forma era la dilaceración. Con relación a la alteración del tamaño, se encontró que los dientes con rizomicri presentan mayor prevalencia, con un 44.4%, a diferencia del autor, quien menciona que la anomalía de tamaño más prevalente en su estudio fue la microdoncia, con el 4,01%.

Valdivia ^24^ encontró, en el grupo de edad de 6 a 9 años, que el 15,7% presentaban anomalías en la forma de los dientes, con 10 varones y 1 mujer afectados. Del mismo modo, el grupo de edad de 10 a 12 años también tuvo una tasa de ocurrencia del 15,7%, con 10 niños que mostraban irregularidades en la forma, incluida 1 mujer. Dichos resultados no guardan relación con el presente trabajo de investigación con relación al grupo etario, debido a que en este se mostró que el grupo etario con mayor prevalencia de anomalías dentarias de tamaño y forma fue el de 11 a 17 años, con un 71,3% (258), y el menos prevalente fue el de 6 a 10 años, mientras que el autor citado menciona en sus resultados que no hubo diferencias entre los grupos etarios, ya que ambos presentaron el mismo porcentaje.

Celis *et al*. ^25^ tuvieron como objetivo investigar la ocurrencia de anomalías dentales (AD) en radiografías panorámicas tomadas en el Centro de Radiología de Lima, entre los años 2020 y 2021. El análisis reveló que el 8,3% se identificó como microdoncia y el 6,1%, como taurodontismo. Se halló que la ocurrencia de estas anomalías es del 20,3% en hombres y el 16,4% en mujeres. La microdoncia se ubicó como la cuarta anomalía más común, con una prevalencia del 3,5% en hombres y el 4,9% en mujeres. El taurodontismo, por su parte, se situó como la quinta anomalía más frecuente, con una prevalencia del 2,3% en hombres y el 3,8% en mujeres. Al examinar la distribución de anomalías dentales por cuadrante, se reveló una dilaceración radicular del 4,2% en el “primer cuadrante, el 2,7% en el segundo cuadrante, el 4,1% en el tercer cuadrante y el 5,6% en el cuarto cuadrante. En el primer cuadrante se observó microdoncia en el 3,5 % de los casos, mientras que en el segundo cuadrante se halló una prevalencia ligeramente mayor, del 3,6%. En contraste, el tercer cuadrante tuvo una tasa mucho más baja, del 0,5%, y el cuarto cuadrante, una del 0,8%. El taurodontismo, por otro lado, se encontró en el 1,1% de los casos, tanto en el primer como en el segundo cuadrante, mientras que el tercer cuadrante tuvo una tasa mayor, del 2,1%, y el cuarto cuadrante, una del 1,7%. Estas estadísticas son exactas y no fueron modificadas de ninguna manera.

A partir de lo señalado, podemos concluir que la alteración dentaria de tamaño y forma presentó mayor prevalencia en los cuadrantes I y II, gracias al estudio del autor, quien presentó resultados similares a los del trabajo de investigación.

## CONCLUSIONES

Existe una mayor prevalencia de anomalías de forma, y los cuadrantes I y II presentan la mayor incidencia de anomalías dentales en alteraciones de número y forma. La alteración dentaria de tamaño se presentó solo en la dentición permanente, mientras que la alteración dentaria de forma se presenta en las piezas permanentes y deciduas. 

Las piezas dentarias que muestran la ocurrencia más frecuente de anomalías dentarias en cuanto a cantidad y tamaño son los incisivos centrales, los incisivos laterales, los caninos y los primeros molares.

## Financiamiento:

Autofinanciado
